# Predicting vesicoureteral reflux outcomes using artificial intelligence: A critical appraisal using APPRAISE-AI

**DOI:** 10.1371/journal.pdig.0001237

**Published:** 2026-02-13

**Authors:** Adree Khondker, Sanchit Kaushal, Jeremy Wu, Naveen Gupta, Jethro CC. Kwong, Tiange Li, Lauren Erdman, Mandy Rickard, Armando J. Lorenzo

**Affiliations:** 1 Division of Urology, The Hospital for Sick Children, Toronto, Ontario, Canada; 2 Division of Urology, Department of Surgery, University of Toronto, Toronto, Ontario, Canada; 3 Temerty Faculty of Medicine, University of Toronto, Toronto, Ontario, Canada; 4 Department of Pediatrics, Cincinnati Children’s Hospital Medical Center, University of Cincinnati, Cincinnati, Ohio, United States of America; Guangdong Provincial People's Hospital, CHINA

## Abstract

Vesicoureteral reflux (VUR) is a common congenital urinary tract anomaly in children, associated with recurrent urinary tract infections (UTIs) and long-term sequelae such as renal scarring and chronic kidney disease. Artificial intelligence (AI) has recently emerged as a promising approach for improving VUR diagnosis, prognosis, and treatment stratification. This narrative review identified studies from the AI-PEDURO repository, a living database of AI applications in pediatric urology, most recently updated in June 2024. Eligible studies employed machine learning methods to predict clinically relevant outcomes of VUR or UTI. Seventeen studies met the inclusion criteria, with common applications including VUR grading from voiding cystourethrograms, prediction of UTI recurrence, spontaneous resolution of VUR, and outcomes following antibiotic prophylaxis or endoscopic injection therapy. Neural networks, tree-based algorithms, and support vector machines were the most frequently used approaches. Using the APPRAISE-AI tool, the median overall study quality was moderate, with strengths in clinical relevance and reporting quality, but persistent weaknesses in methodological conduct, robustness, and reproducibility. AI applications in VUR demonstrate strong potential to enhance diagnostic accuracy, personalize treatment, and predict outcomes; however, most published models remain of low to moderate quality. Adoption of standardized reporting frameworks and multi-institutional collaboration will be essential for improving rigour and accelerating clinical translation.

## Author summary

Vesicoureteral reflux (VUR) is a condition in children where urine flows backwards from the bladder toward the kidneys. This can increase the risk of urinary tract infections and, in severe cases, lead to kidney damage later in life. Doctors often face challenges in deciding which children need treatment and how best to monitor the condition. Recently, artificial intelligence (AI) has been explored as a tool to improve diagnosis and guide care. In this review, we examined all available studies that applied AI to VUR, including its use in interpreting imaging, predicting which children might benefit from specific treatments, and identifying those at higher risk for complications. We found that while many studies show promising results, most were small in size, not tested across different hospitals, and often lacked the detail needed for other researchers to reproduce the work. Despite these challenges, AI has the potential to make VUR diagnosis more consistent and treatment more personalized. Future research should focus on larger, collaborative studies and clearer reporting to determine how AI can best support children, families, and clinicians in managing this condition.

## Introduction

Vesicoureteral reflux (VUR) is among the most common congenital malformations of the urinary tract in children [1]. It is associated with recurrent urinary tract infections (UTIs) and long-term sequelae, including reflux nephropathy, hypertension, proteinuria, and, in severe cases, chronic kidney disease (CKD) or end-stage renal disease (ESRD) [1,2]. While management of symptomatic VUR is aimed at preventing UTIs and renal scarring, the clinical decision-making in asymptomatic cases remains complex [3]. Predicting spontaneous resolution is particularly challenging; although multivariable tools incorporating a multitude of clinical factors have been developed, such as nomogram tables, the VUR index, and the distal ureteral diameter ratio [1,4–7].

Artificial intelligence (AI) has rapidly emerged as a promising tool in pediatric urology, with VUR being the most investigated topic to date [8,9]. AI models have been developed to predict UTI recurrence, standardize VUR grading, and estimate surgical success, among other outcomes [8–10]. The appeal of AI is clear, given limitations of existing prediction tools and controversies in VUR management. However, despite increasing enthusiasm, the quality and applicability of AI models on this topic have yet to be evaluated.

In this narrative review, we leverage the AI-PEDURO repository to identify published studies and AI models applied to VUR. Using the APPRAISE-AI tool [11], we also evaluate the methodological and reporting quality of their approach, with the intention of guiding future research and clinical translation.

## Methods

### Study selection, eligibility criteria, and data extraction

Studies that have included AI models on VUR and UTI were selected from the AI-PEDURO repository. This is a living online repository of AI models in pediatric urology, with the most recent systematic search performed in June 2024. Further information regarding the protocol and search strategy for the repository is documented in separate publications [8,12]. The search strategy that was utilized for this appraisal is provided in the S1 Table.

The criterion for inclusion is the prediction of clinically relevant outcomes relevant to VUR and UTI utilizing machine learning (ML) methods. The outcomes of interest include risk of breakthrough or febrile UTI, resolution of VUR, need for surgery, and renal complications. AI models included tree-based models, support vector machines (SVM), artificial neural networks, and deep learning methods.

Data extraction was performed independently by two reviewers (JW, NG) and verified by two separate authors (AK, JK). Discrepancies in data reporting were solved through consensus. The following details were extracted from studies and qualitatively synthesized into a table: study details, population characteristics, VUR severity, UTI frequency, model inputs/outputs, performance metrics, and clinical usability. Meta-analysis was not performed within the context of this narrative review due to significant expected heterogeneity in study outcomes and expected paucity of high-quality studies.

### Critical appraisal of models

Studies were critically appraised using the APPRAISE-AI tool, which comprises 24 methodological and reporting items that reflect best practices in AI. These correspond to six domains: clinical relevance, data quality, methodological conduct, robustness of results, reporting quality, and reproducibility. Three raters with ML expertise individually assessed each article for APPRAISE-AI score, and disagreements were resolved by consensus. Overall scores can range from 0 to 100 points, corresponding to the following levels of study quality: scores from 0 to 19 indicate very low quality, 20 to 39 indicate low quality, 40 to 59 indicate moderate quality, 60 to 79 indicate high quality, and 80 to 100 indicate very high quality. Points were weighted toward title and introduction (items 1–3, of 3 points), methods (items 4–12, of 51 points), results (items 13–19, of 27 points), discussion (items 20–22, of 8 points), disclosures (item 23, of 1 point), and transparency (item 24, of 10 points). APPRAISE-AI was selected over other similar guidelines, such as PROBAST-AI, as it provides a quantitative metric for model quality [13].

Three raters (AK, JW, NG) performed APPRAISE-AI on all included studies, and the majority decision was taken as the result for each measure. In cases of disagreement between all three reviewers, a fourth rater was involved (JK).

## Results and discussion

### Study characteristics

A total of 17 studies met the inclusion criteria and were extracted from the AI-PEDURO repository in June 2024 (Table 1), and a PRISMA flow diagram is provided in S1 Fig. Of these studies, 7 (41%) were published in 2020 or earlier, while the remaining 10 (59%) were published in 2021–2024. The most common topics of study were determining the VUR grade using images from VCUGs (35%), predicting the recurrence of UTI (18%), and predicting the chance of VUR resolution (12%). The studies were retrieved from 10 unique peer-reviewed journals, with the Journal of Pediatric Urology (5 studies, 29%) and Journal of Urology (3 studies, 18%) being the most common journals in which studies were published. Of the 17 studies, 5 (29%) used multiple ML algorithms. The most frequently employed algorithms were neural networks (9 studies, 52.9%), tree-based models (7 studies, 41%), and SVM (3 studies, 18%).

**Table 1:** Study characteristics and performance metrics of studies focused on VUR.

Study	Objective	AI Approach	Data Source(s)	Model Input Variables	Model Outcome and Performance [Validation approach]	Model Quality (APPRAISE-AI)
Arlen (2016) [14]	To predict the probability of breakthrough fUTI in children with primary VUR.	2-hidden node neural network	Institutional series (384 VCUGs)	Age, gender, laterality, percentage PBC at VUR onset, VUR grade right/left, VUR onset right/left (filling or voiding), complete ureteral duplication, number of UTIs prior to VUR diagnosis (2 vs. <2), history of fUTI, and history of BBD.	Predict breakthrough febrile UTI occurrence: AUC 0.76 [Holdout validation]	Low
Bertsimas (2021) [15]	To predict which patients with VUR are most likely to benefit from continuous antibiotic prophylaxis	Optimal classification trees	Multi-institutional trial dataset (RIVUR, 607 patients)	Race, gender, VUR grade, serum creatinine, prior UTI symptoms, weight percentiles	Risk of recurrent UTI: AUC of 0.82 [Holdout validation]	Moderate
Dubrov (2021) [16]	To predict the outcomes of a single endoscopic injection of DxHA for correction of VUR	Multilayer ANN (multilayer perceptron) with two hidden layers and a sigmoid function of neuronal activation	Multi-institutional series (582 children, 783 ureteric units operated on)	VUR grade, age, sex, presence of ureteral duplication, ureteral dilatation index	To predict outcome of DxHA injection treatment: AUC of 0.77, correct prognosis 74.5%, sensitivity 85.5%, specificity 65.3% Clinic 1: AUC of 0.72, correct prognosis 69%, sensitivity 91%, specificity 30.2% Clinic 2: AUC of 0.8, correct prognosis 78%, sensitivity 70%, specificity 82% [Holdout split]	Moderate
Eroglu (2021) [17]	To determine VUR grade using images from VCUGs	Hybrid CNN (+ K-nearest neighbors or + SVM)	Institutional series (1228 images)	Raw VCUG images	To predict each normal and each VUR grade: AUC of 0.99, and accuracy of 97% [Holdout validation]	Low
Ganapathy (2023) [18]	To predict renal scarring in pediatric population with VUR	Logistic Regression, Discriminant Analysis, Bayesian Logistic Regression, Naïve Bayes, Decision Tree	Institutional series (94 children)	Kidney injury molecule-1 (KIM-1), Neutrophil gelatinase–associated lipocalin (NGAL), Urinary creatinine, Ratios of NGAL and KIM-1 to urinary creatinine	To predict the presence of renal scarring: Logistic Regression: AUC = 0.83 Discriminant Analysis: AUC = 0.83 Bayesian Logistic Regression: AUC = 0.83 Naïve Bayes: AUC = 0.81 Decision Tree (C5.0): AUC = 0.83 [Unspecified validation]	Low
Kabir (2024) [19]	To determine VUR severity using quantitative features extracted from VCUG image	6 classification algorithms (MLP, Tree-based, SVM, etc.)	Online image scraping (113 VCUG images)	VCUG extracted features: max ureter width, ureter tortuosity, UPJ width, UVJ width, ureter curvature, ureter area, pelvicalyceal outline length, pelvicalyceal region area, pelvicalyceal outline curvature	Assessing binary VUR grade (low [I-III] vs. high [IV-V]). MLP: AUC of 0.96, F1-score of 0.91 SVM: AUC of 0.95, F1-score of 0.90 [leave-one-out analysis]	Moderate
Keskinoglu (2020) [20]	To determine a diagnosis of VUR versus UTI	ANN	Institutional series (611 children)	39 variables (clinical, laboratory, and US findings)	VUR/UTI: AUC of 0.81, and precision of 0.78 [cross validation]	Low
Khondker (2021) [21]	To predict high-grade VUR from quantitative features annotated from VCUGs	RF	Web scraping (41 renal units), institutional series (44 renal units)	Ureter tortuosity, UPJ width, UVJ width, and maximum ureter width on VCUG	High-grade VUR: accuracy of 0.83, AUC of 0.90 [Leave-one-out analysis]	High
Khondker (2022) [22]	To predict VUR grade from quantitative features annotated from VCUGs	RF	Multi-institutional series (1248 renal units, from 4 institutions, 1 web-scraped dataset, 1 community dataset)	Ureter tortuosity, proximal ureter width, distal ureter width, and maximum ureter width on VCUG	Grade II vs III vs IV vs V: AUC of 0.75-0.94 (training), AUC of 0.72-0.91 (external). [Cross validation and external validation]	High
Knudson (2007) [23]	To predict the chance of early VUR resolution.	Linear SVM	Institutional Series (205 children)	Age, gender, presenting symptoms, reflux grade, laterality, whether reflex occurred during filling or voiding, initial bladder volume at onset, presence of complete ureteral duplication	Prediction of resolution 1 year after diagnosis: AUC of 0.819. Prediction of resolution 2 years after diagnosis: AUC of 0.86 [Holdout validation]	Low
Li (2024) [24]	To develop and validate a deep-VCUG with ensemble learning for automatic VUR grading from VCUG images	CNN, Ensemble learning	Multi-institutional series (1948 images, from 5 institutions)	512 × 512 pixels VCUG images	Unilateral VUR: AUC of 0.96 (internal), AUC of 0.94 (external); Bilateral VUR: AUC of 0.96 (internal), 0.92 (external) [split-sample validation, external validation]	High
Logvinenko (2015) [25]	To predict VUR grade from renal and bladder US findings on the same day	ANN	Institutional series (2259 children)	RBUS findings, sex, age, circumcision status (in boys), febrile UTI, first (vs. recurrent) UTI	Any VUR: AUC of 0.69 VUR grade > II: AUC of 0.67 VUR grade > III: AUC of 0.79 [Unclear validation method]	Moderate
Seckiner (2008) [26]	To predict the resolution of VUR	ANN	Institutional series (145 ureteric units)	Age, sex, the cause and grade of VUR, the affected ureter, the type of treatment, existence of renal scar on DMSA scan, follow-up times, the number of injection	VUR resolution: accuracy of 0.98 VUR improvement: accuracy of 1.0 VUR persistent or worse: accuracy of 0.92 [Holdout validation]	Moderate
Serrano-Durba (2004) [27]	To predict the results of endoscopic treatment for VUR	ANN	Institutional series (261 ureteric units)	Age, sex, cause/grade of VUR, type/number of implanted substance, number of treatments, affected ureter, endoscopic findings, type of cystography	Success of endoscopic treatment: AUC of 0.77 [Holdout validation]	Low
Estrada (2019) [28]	To predict the probability of recurrent UTI and associated VUR after initial UTI but before VCUG	Optimal classification Trees, RF, gradient-boosted trees	Multi-institutional trial dataset (RIVUR, 305 patients; CUTIE, 195 patients)	Age, gender, race, weight, antibiotic resistance in urine culture, urine protein, dysuria, medications, antibiotics in last 6 months, blood pressure	VUR-associated recurrent UTI: AUC of 0.76 [Holdout and cross validation]	High
Lee (2022) [29]	To predict the recurrence of UTI after ^99m^Tc-DMSA renal scan	CNN	Institutional series (180 patients)	Pre-processed ^99m^Tc-DMSA images	Recurrent UTI: accuracy of 0.91 [Cross validation]	Moderate
Wang (2024) [30]	To reliably predict dilating VUR from early postnatal US in patients with hydronephrosis	Optimal Classification Tree (OCT)	Institutional series (280 patients, 530 renal units)	Patient demographics (age at US, sex, kidney laterality) and UTD features (primary antero-posterior diameter, central calyceal dilation, peripheral calyceal dilation, parenchymal thickness, parenchymal appearance, distal ureteral dilation)	Dilating (>Gr3) VUR: AUC of 0.81 (95% CI: 0.73-0.88) [Holdout and cross validation]	Moderate

### Model quality assessment and appraisal

**Fig 1:**
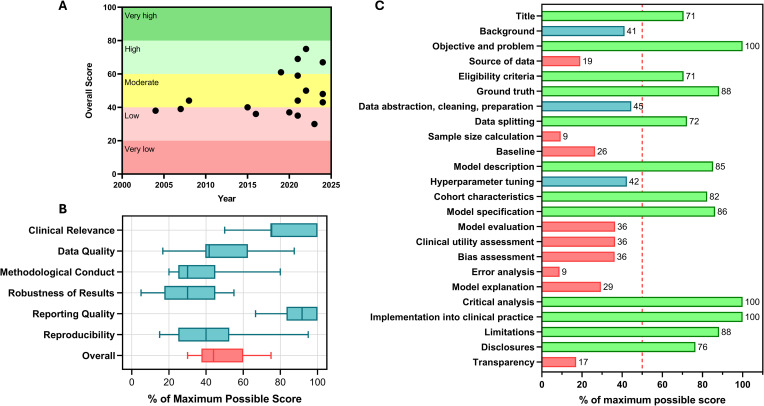
APPRAISE-AI criteria for overall group of studies. **(A)** Overall APPRAISE-AI score over time, **(B)** Model quality by APPRAISE-AI domain, and **(C)** proportion of studies meeting criteria with sufficient reporting.

The APPRAISE-AI tool was utilized to assess model quality across all studies, and the intraclass correlation coefficient for all categories was between 0.88 and 1.0 among raters. The study quality was low in 6 studies (35%), moderate in 7 studies (42%), and high in 4 studies (24%). Overall, study quality has improved over time, as evidenced by the general upward trend of overall APPRAISE-AI scores in Fig 1A. The strongest APPRAISE-AI domains were clinical relevance and reporting quality, while the weakest were methodological conduct, robustness of results, and reproducibility (Fig 1B). The most missed areas were sample size calculation (91%), error analysis (91%), transparency (83%), and source of data (81%) (Fig 1C).

### Improving VUR diagnosis and severity assessment

There remains a high degree of subjectivity in assigning VUR grades using VCUG due to discordance among expert readers using the International Reflux Study Group (IRSG) classification system, with agreement as low as 50%–60% [31–33]. Numerous studies sought to address this concern by applying AI to VCUG interpretation. One novel approach was the Deep-VCUG model developed by Li and colleagues [24], a deep learning model that utilizes deep CNNs and ensemble learning to automate VUR grading on VCUG. The model achieved excellent discrimination for both unilateral (AUC on internal dataset: 0.96; AUC on external dataset: 0.94) and bilateral VUR detection (AUC on internal dataset: 0.96; AUC on external dataset: 0.92). The tool also improved clinician performance, increasing average AUCs for junior and senior readers and more than doubling inter-observer agreement. On APPRAISE-AI, this study was rated high quality, supported by adherence to STREAM-URO reporting practices, multicentre validation, and open-source code release [34].

Similarly, Eroglu and colleagues [17] trained a hybrid-based mRMR (Minimum Redundancy Maximum Relevance) model using a CNN (Convolutional Neural Network) to diagnose and grade VUR, utilizing 1228 raw VCUG images. Their model proved successful, reporting an AUC of 0.99 and an accuracy of 97% in their dataset; however, the sample size was small, limiting validity.

More recently, Khondker and colleagues [21] used a random-forest classifier to detect high-grade VUR (AUC 0.83; accuracy 0.90). In an independent, multi-institutional validation cohort of 1,492 kidneys/ureters, the same group combined clinical features with VCUG-derived measurements (e.g., ureteral tortuosity; proximal, distal, and maximal ureteral width) to discriminate among grades II–V with AUCs ranging from 0.72 to 0.91 [22]. Reliability improved 3.6-fold over traditional gold standard reporting by radiologists and APPRAISE-AI adjudication deemed the work high quality, aided by full methodological transparency and release of the complete code base. Kabir and colleagues [19] extracted additional features from VCUG and classified cases by severity using a multilayer perceptron, achieving an AUC of 0.96 and F1-score of 0.91 within a sample of online web-scraped images.

Collectively, AI-based models for VUR grading and detection demonstrated consistently high discriminatory performance. Imaging-based tasks, particularly automated interpretation of VCUG, represented the most mature application of AI in this domain, likely reflecting the structured nature of the input data and the well-defined classification targets. However, performance metrics varied substantially depending on the validation strategy. Models evaluated exclusively on internal datasets tended to report the highest AUC. This suggests a degree of optimism bias rather than model failure and highlights the importance of validation context when interpreting discrimination metrics. Collectively, these results indicate that AI can meaningfully improve consistency and reliability of VUR grading, but that high AUCs on their own should not be interpreted as evidence of clinical readiness.

### Guiding the decision to perform additional imaging

The timing and necessity of diagnostic imaging remain debated; although VCUG is the diagnostic gold standard, it is invasive, entails exposure to ionizing radiation, and can be distressing for families [33]. To inform selective imaging, an optimal classification tree (OCT) using clinical and laboratory variables was developed from the RIVUR and CUTIE studies to predict UTI recurrence associated with VUR [28]. The model supported individualized decisions after an index UTI and identified children most likely to benefit from VCUG. On APPRAISE-AI, the study scored highly based on sample validation, model development, and transparency. Wang and colleagues [30] similarly employed an OCT to predict dilating VUR (grade ≥3) from early postnatal ultrasound in children with hydronephrosis, combining demographic and sonographic features (gender, ureteral dilation, parenchymal appearance/thickness, central calyceal dilation) and achieving an AUC of 0.81.

Separately, Logvinenko and colleagues [25] trained an ANN to predict VUR from renal–bladder ultrasound (RBUS) prior to VCUG in 2,259 patients; performance was modest (AUC 0.69), underscoring the limited predictive value of RBUS abnormalities alone—even with AI. Keskinoglu and colleagues [20] reported an AUC of 0.81 using 39 clinical, laboratory, and ultrasound variables in children with uncomplicated UTIs, but specificity was poor, and this limits the clinical utility of the model.

Across studies evaluating AI to guide selective imaging, model performance was more variable than that observed for VCUG grading, with AUCs ranging from modest to good discrimination. This variability reflects both heterogeneity in outcome definitions and the inherent challenge of predicting VUR presence or severity using indirect clinical and sonographic markers. Models derived from large, well-characterized cohorts, particularly those leveraging randomized trial data (RIVUR/CUTIE) demonstrated more robust and clinically interpretable performance, suggesting that data provenance plays a critical role in model reliability.

### Selecting high-risk patients for continuous antibiotics or surgery

One of the biggest challenges in VUR management is identifying and individualizing patients who will benefit from the treatment options available. For example, while continuous antibiotic prophylaxis (CAP) is considered standard of care for all grades of reflux, it should be reserved for selected high-risk patients due to growing concerns about antibiotic resistance, low adherence, and potential effects on the microbiome [1,35]. To address this, Bertsimas and colleagues applied OCTs to the RIVUR dataset, which included 607 children across 19 U.S. centers [15]. They developed a predictive model to identify patients with VUR who are most likely to benefit from CAP while minimizing its use in those with the least need. Their model achieved an AUC of 0.82, demonstrating that treating only 40% of patients could yield population-level benefits of similar equivalence to treating all patients. The study was rated as moderate quality using the APPRAISE-AI framework, and strengths included the use of a robust, well-characterized randomized trial dataset, clearly defined outcome measures, and detailed reporting of predictors and model performance.

Another treatment option for pediatric VUR patients is endoscopic dextranomer/hyaluronic acid copolymer (DxHA) injection therapy, which has emerged as a minimally invasive and well-tolerated treatment modality compared to open surgery [33]. Dubrov and colleagues [16] applied a multi-layer ANN to predict factors related to outcomes following injection. Their model demonstrated an accuracy rate of 74.5%, with an AUC of 0.77, a sensitivity of 85.5%, and a specificity of 65.3%. Previously, Serrano-Durba and colleagues [27] demonstrated that their ANN model was effective in predicting the success of endoscopic treatment for patients with VUR, with an AUC of 0.77 as well.

Because both surgery and prolonged CAP can carry long-term consequences, predicting spontaneous resolution is clinically valuable. Knudson and colleagues [23] used a linear SVM to predict resolution in 205 children at 1 and 2 years (AUCs of 0.819 and 0.86, respectively). Seckiner and colleagues [26] developed an ANN based on demographic and clinical variables that achieved an accuracy of 0.98. Beyond primary VUR disease manifestation, there is growing interest in predicting and preventing recurrence of UTI in pediatric patients with VUR. The ^99m^Tc-DMSA scan is often used for the detection of scarring following a febrile UTI. In their institutional series of 180 patients, Lee and colleagues [29] successfully developed a CNN model using pre-processed ^99m^Tc-DMSA images to predict the recurrence of UTI with an AUC of 0.91. This is a valuable finding, given that increased diagnostic accuracy, greater than that of conventional methods, allows us to better risk-stratify patients at high risk of recurrent UTIs that may require further treatment, such as CAP.

AI models designed to guide treatment decisions in VUR demonstrate moderate to good discrimination, with AUCs typically ranging from 0.77 to 0.86. These outcomes reflect the inherent complexity of treatment-response prediction, which is influenced by longitudinal factors such as adherence, infection recurrence, and clinician practice patterns. Notably, studies developed using prospective or randomized trial datasets achieved clinically meaningful performance despite not always reporting the highest AUCs, emphasizing that methodological rigor and alignment with decision-making thresholds may be more relevant than marginal improvements in discrimination.

### Predicting complications and disease prognosis

In addition to personalizing treatment options for patients with VUR, we identified several AI models that have been developed to predict patients at increased risk of developing long-term kidney outcomes, such as renal scarring, without needing a nuclear medicine study. Ganapathy and colleagues [18] demonstrated that their decision tree (AUC of 0.83) and Naïve Bayes models (misclassification rate of 20.2%) accurately predicted renal scarring using a combination of biomarkers, outperforming individual biomarkers alone. However, the study was determined to be of overall low quality.

Much attention has also been placed on predicting the risk of breakthrough febrile urinary tract infection (fUTI) given its influence on determining whether surgical correction is necessary for VUR. Arlen and colleagues [14] developed a computational model to predict which children are at risk of fUTI, incorporating valuable clinical information such as VCUG imaging data, a history of multiple infections/fUTI, and a history of bladder and bowel dysfunction (BBD). Their model demonstrated an AUC of 0.76, and they additionally published a web-based prognostic calculator to aid pediatric urologists.

Prognostic AI models predicting complication risk and disease progression demonstrated heterogeneous performance, with reported AUCs spanning from 0.76 to 0.83. Nevertheless, these models identified clinically plausible associations between imaging features, infection history, and adverse outcomes, suggesting that AI may play a future role in long-term risk stratification.

## Outlook on the intersection of VUR and AI

VUR and UTI are the most common topics in pediatric urology to which AI has been applied. In this narrative review, we identified 17 models that have been used to address various clinical questions, including predicting the risk of infection, VUR resolution, improving radiographic grading, and predicting complications. The most researched topics were improving the diagnosis and management of VUR using VCUG, as well as predicting the spontaneous resolution and recurrence of UTI. Overall, AI applications in VUR demonstrate a clear gradient in performance across clinical tasks. Imaging-based diagnostic models show the most consistent and highest discrimination, while prognostic and treatment-selection models exhibit greater variability reflecting increased clinical and temporal complexity. Across all domains, internally validated studies tend to report higher AUCs than externally validated models, underscoring the influence of validation context and the potential for optimism bias. Importantly, high AUCs do not equate to clinical readiness; factors such as reproducibility, calibration, interpretability, and workflow integration remain critical determinants of translational potential.

We used the APPRAISE-AI tool to assess the methodological rigour and reporting quality of these studies and found that while the overall quality of models was moderate and has improved over time, only 25% of the studies were deemed to be high quality. Our work revealed a consistent pattern: studies performed best in the domains of clinical relevance and reporting quality, while methodological conduct, robustness of results, and reproducibility received the lowest scores. This mirrors findings from other evaluations of AI research in urology, including the recent living scoping review of pediatric urology AI models [12,36]. Common challenges included small sample sizes, which increase the risk of overfitting and limit external validity. As highlighted by Wang and colleagues, methodological practices such as bootstrapping, improved data-splitting strategies, cross-validation, and multicenter collaborations are crucial for strengthening model performance and generalizability [9]. Reproducibility remains another key limitation. As Kwong and colleagues emphasize, improving reproducibility requires practices that are not yet standard in clinical research, including comprehensive model description, hyperparameter tuning, and transparency in code and dataset sharing [36]. These practices, while fundamental to AI, are not routinely required by journals and therefore remain underreported. To ensure that AI models translate into meaningful improvements in clinical outcomes, future research should adopt standardized frameworks for development and reporting, such as DECIDE-AI and STREAM-URO [34,37].

Certain studies met high-quality criteria can serve as an example for improved reporting. Khondker and colleagues (2021, 2022) received higher scores for including comparison against current standard of care models compared to their ML models, including external validation, and using the STREAM-URO framework to ensure data sources were appropriately reported [21,22]. Meanwhile, both Estrada and colleagues (2019) and Li and colleagues (2024) clearly described data handling and provided a bias assessment of their model to achieve high quality [24,28].

### Limitations

Some limitations need to be addressed in our narrative review. While we included 17 studies that explored the use of AI for predicting VUR outcomes, we did not conduct a systematic review or meta-analysis, which limits our ability to synthesize the outcomes. This was decided due to the heterogeneity of models and outcomes being measured. Additionally, we utilized APPRAISE-AI to evaluate model quality, which was published after the publication dates of some of the included studies, and best practices in AI have evolved over time.

## Conclusion

AI has been applied to multiple aspects of VUR care, including diagnostic imaging, risk prediction, treatment selection, and prognosis. To date, most studies remain exploratory, with limited external validation, modest sample sizes, and variable methodological rigour. Despite these challenges, AI shows considerable promise in improving the consistency of VUR grading, individualizing treatment decisions, and refining prognostic counseling. Future research should focus on multicenter prospective studies, transparent reporting, and integration into clinical workflows to determine the true impact of AI on patient outcomes and whether it offers benefits beyond current standard practice.

## Supporting information

S1 Fig PRISMA flow chart for included VUR studies from AI-PEDURO repository. (DOCX)

S1 Table Search strategy for AI-PEDURO repository from Embase Classic+Embase 1947–2024 June 21. (DOCX)
