# Supplementary material for: Predicting vesicoureteral reflux outcomes using artificial intelligence: A critical appraisal using APPRAISE-AI
Source: PLOS Digit Health. 2026 Feb 13;5(2):e0001237. doi: 10.1371/journal.pdig.0001237 (PMC12904409; doi:10.1371/journal.pdig.0001237)
Supplement: S1 Fig — (DOCX) [file pdig.0001237.s001.docx]

## S1 Fig: PRISMA flow chart for included VUR studies from AI-PEDURO repository.
