# Supplementary material for: Predicting vesicoureteral reflux outcomes using artificial intelligence: A critical appraisal using APPRAISE-AI
Source: PLOS Digit Health. 2026 Feb 13;5(2):e0001237. doi: 10.1371/journal.pdig.0001237 (PMC12904409; doi:10.1371/journal.pdig.0001237)
Supplement: S1 Table — (DOCX) [file pdig.0001237.s002.docx]

## S1 Table: Search Strategy for AI-PEDURO repository from Embase Classic+Embase 1947 to 2024 June 21.

| **#** | **Searches** | **Results** |
| --- | --- | --- |
| 1 | exp child/ or exp pediatrics/ or child*.ti,ab. or infan*.ti,ab. or (baby or babies).ti,ab. or exp adolescent/ or adolescen*.ti,ab. or (pediatric*1 or paediatric*1).ti,ab. or (neonat* or newborn*).ti,ab. | 5607953 |
| 2 | exp urology/ or exp child urology/ | 53835 |
| 3 | exp hydronephrosis/ or exp vesicoureteral reflux/ | 47658 |
| 4 | exp urodynamics/ or exp obstructive uropathy/ | 72909 |
| 5 | exp spinal dysraphism/ | 16851 |
| 6 | exp urinary tract infection/ | 158349 |
| 7 | exp pyeloplasty/ | 5215 |
| 8 | exp nephroblastoma/ | 21337 |
| 9 | exp hypospadias/ | 13272 |
| 10 | exp urolithiasis/ or exp nephrolithiasis/ | 85029 |
| 11 | exp artificial intelligence/ or exp machine learning/ | 527258 |
| 12 | (neural network or support vector machine or multilayer perceptron or neural network or random forest or lasso or ridge or kernel or bayesian network or classification tree or regression tree or vector machine or nearest neighbor or probability estimation tree or elastic net or ensemble or penalized or regularized or bagging or boosting or fuzzy or bayes or deep learning).ti,ab. | 358863 |
| 13 | 1 and (2 or 3 or 4 or 5 or 6 or 7 or 8 or 9 or 10) and (11 or 12) | 473 |
